# Effect of host shift on the gut microbes of *Bactrocera cucurbitae* (Coquillett) (Diptera: Tephritidae)

**DOI:** 10.3389/fmicb.2023.1264788

**Published:** 2023-11-21

**Authors:** Zhenya Tian, Lixiang Chen, Guangmei Chen, Jingjing Wang, Chao Ma, Yan Zhang, Xuyuan Gao, Hongsong Chen, Zhongshi Zhou

**Affiliations:** ^1^College of Agriculture, Guangxi University, Nanning, China; ^2^State Key Laboratory for Biology of Plant Diseases and Insect Pests, Institute of Plant Protection, Chinese Academy of Agricultural Sciences, Beijing, China; ^3^National Nanfan Research Institute, Chinese Academy of Agricultural Sciences, Sanya, China; ^4^Guangxi Key Laboratory for Biology of Crop Diseases and Insect Pests, Institute of Plant Protection, Guangxi Academy of Agricultural Sciences, Nanning, China

**Keywords:** *Bactrocera cucurbitae*, gut microorganisms, original host, different hosts, host transfer

## Abstract

**Background:**

Gut microbes play an important role in the adaptation of insects. Polyphagous insects usually undergo changes in gut microbiota after host shift. The *Bactrocera cucurbitae* have a wide range of hosts, but the dynamic of gut microorganisms during host shift have not been thoroughly investigated. To understand the role of gut microbes in insect adaptation, it is necessary to study the dynamics of insect gut microorganisms during host transfer.

**Methods:**

Using *Bactrocera cucurbitae* (Coquillett) (Diptera: Tephritidae) and its four hosts as study subjects, we investigated the dynamics of gut microbes during host transfer and the effects of different hosts on the gut microbial composition of *B. cucurbitae*.

**Results:**

The results showed that the Chao1 index of *B. cucurbitae* decreased significantly during host transfer, and the intestinal microorganisms were significantly affected by the original host, host, and generations. Furthermore, predicated changes in the abundance of secondary metabolite pathways after host transfer suggested that microorganisms may play an important role in the degradation of secondary metabolites, among which *Providencia* and *Morganella* have important functions in the gut of *B. cucurbitae*.

**Conclusion:**

This implied that microorganisms play a function in the host transfer process of *B. cucurbitae* and may be an important cofactor in the adaptation of *B. cucurbitae* to different hosts and environments, providing new research ideas for the future control of *B. cucurbitae*.

## 1 Introduction

Microbes are widespread in nature and capable of forming stable symbiotic relationships with a wide range of organisms, playing a very important role within them (Peixoto et al., 2021; Sieber et al., 2021; Koga et al., 2022). Many microorganisms have been shown to positively influence their hosts and co-evolve during symbiosis (Morrison et al., 2009; Shiffman et al., 2017).

Under particular circumstances, the gut often generates insect-specific gut microbial communities that serve as a crucial interface between insects and their surrounding environment (Engel and Moran, 2013). Insect gut microbes play an important role in host growth, development, reproduction, and nutrient metabolism (Shin et al., 2011; Chouaia et al., 2012; Hansen and Moran, 2014), as well as in protecting hosts against natural enemies, pathogens, and viruses (Engel and Moran, 2013; McLean and Godfray, 2015). These microbes also aid the host in metabolizing secondary metabolites in food and pesticides in the environment (Ceja-Navarro et al., 2015; Pang et al., 2018; Zhang et al., 2020; Sato et al., 2021; Zhang S. et al., 2022). In addition to selection pressure by the natural environment, human activity affects the evolution of insects (Goulson, 2020; Soroye et al., 2020; Weidenmüller et al., 2022). In this situation, insects must adapt quickly to deal with the shifting environmental forces; however, their evolutionary processes are sluggish. In contrast, symbiotic bacteria can react to external changes more quickly, giving hosts sufficient time to adapt (Rosenberg and Zilber-Rosenberg, 2018). In other words, the presence of gut microorganisms can also enhance pest adaptation, making them more harmful and pest control harder to implement. As an example, *Citrobacter* sp. (*CF*-BD) increased the resistance of *Bactrocera dorsalis* (Hendel) to trichlorfon, and a phosphohydrolase gene that degrades trichlorfon was identified in *CF*-BD (Cheng et al., 2017). Hence, a direction with significant potential for gut microbial research is the manipulation of particular gut microbes for efficient management of pest infestations (Berasategui et al., 2016). Insect gut bacteria are significantly influenced by environmental factors, such as location, food type, and growth conditions (Liu et al., 2018; Yu et al., 2021; da Silva et al., 2022; Hendrycks et al., 2022; Palmer-Young et al., 2022). During the larval stages, hosts can profoundly influence key adaptive traits in adults during development and are an important manifestation of insect–host interactions (Portman et al., 2015; Pocius et al., 2022). However, for polyphagous insects, feeding on different hosts usually affects their gut microbiota (Yang et al., 2022). For example, the gut microbiota changes significantly when *Plutella xylostella* is transferred from radishes to peas and grows until the 17th generation (Yang et al., 2020). Consequently, a novel perspective on pest control could be obtained by studying the structure of the microbial community in insect guts and its dynamics during host transfer.

Different plants can produce different secondary metabolites, such as alkaloids, organic acids, amines, phenols, quinones, etc. These secondary metabolites usually have unique insecticidal activities and can be used as plant-source pesticides to kill insects (Mithöfer and Boland, 2012; Wang et al., 2018; Divekar et al., 2022). Studies have shown that gut bacteria of herbivorous insects play an important role in the process of metabolizing secondary metabolites contained in plants, and the composition characteristics of gut bacteria of herbivorous insects are also influenced by secondary metabolites in food (Mason and Raffa, 2014; Berasategui et al., 2017; Cheng et al., 2018). An *Acinetobacter* sp., which was found in the gut of seed-damaging *Curculio chinensis* of the *Camellia* species, imparted host-specific degradation of the secondary metabolite teasaponin in oilseeds and decreased the deleterious effects of feeding on *Camellia* species (Zhang et al., 2020; Zhang S. et al., 2022). Therefore, the dynamic change of intestinal bacteria composition is also an important manifestation of insect adaptation to the environment.

*Bactrocera cucurbitae* (Coquillett) (Diptera: Tephritidae) is an important pest with a wide range of hosts, establishing large infestation areas outside its native range; it is thought to have originated in India and is now widely distributed in most parts of the tropics, subtropics, and temperate zones (Virgilio et al., 2010; De Meyer et al., 2015; Delatte et al., 2019). The classification of *Bactrocera (Zeugidacus) cuurbitae* versus *Zeugodacus (Zeugodacus) cucurbitae* remains controversial, and we refer to the more common combination of *Bactrocera (Zeugidacus) cuurbitae* to classify melon fly (Krosch et al., 2012; Virgilio et al., 2015). *B. cucurbitae* can affect up to 39 families and more than 130 plant species, including cucurbit crops, such as the bitter gourd (*Momordica charantia* L.), *Citrullus lanatus* (Thunb.), and *Cucumis melo* L. as the main hosts (Mcquate et al., 2017). Owing to *B. cucurbitae*’s high adaptability and propensity for reproduction, traditional control methods tend to be ineffective (Choudhary et al., 2021). Studies have been conducted to characterize the gut microorganisms of the *B. cucurbitae* at different developmental stages (Choudhary et al., 2021). However, as a polyphagous insect, the dynamics of gut microorganisms during the transition between its hosts have not been thoroughly investigated.

This study aimed to enrich our understanding of the gut flora of *B. cucurbitae* by examining the dynamics of gut microorganisms in three generations of larvae after interconversion between four different hosts. Further, this study provides a new perspective for studying the relationship between *B. cucurbitae* and its host plants.

## 2 Materials and methods

### 2.1 Insect rearing and sampling

The larvae of *B. cucurbitae* were collected from melon fields in Nanning, Guangxi Province, China, raised in greenhouses where temperature, humidity, and light can be controlled (T = 25 ± 5°C, relative humidity = 70 ± 10%, photoperiod L:D = 14:10). The larvae were raised in the corresponding melons until they mature. Then, fine sand with appropriate humidity was provided for the larvae to pupate. After the *B. cucurbitae* emerges, it was transferred to a 30 * 30 * 30 breathable cage for breeding. There were about 200 adults in the cage, and water and feed (yeast powder: white sugar = 1:2) were provided to feed the adults. Chieh-qua (*Benincasa hispida* Cogn. var. Chiehqua How), cucumbers (*C. sativus*), loofah (*Luffa aegyptiaca* Mill.), and bitter gourd (*M. charantia*) were used to rear the larvae for more than 16 consecutive generations to obtain populations of melon fruit flies growing on each of the four hosts. Subsequently, larvae from melons that differed from the original host were reared separately for three consecutive generations. Chieh-qua population transferred to cucumber, loofah, and bitter melon was recorded as BTC, BTL, and BTM, respectively; cucumber population transferred to Chieh-qua, loofah, and bitter melon was recorded as CTB, CTL, and CTM, respectively; loofah population transferred to Chieh-qua, cucumber, and bitter melon was recorded as LTB, LTC, and LTM, respectively; and bitter melon population transferred to Chieh-qua, cucumber, and loofah was recorded as MTB, MTC, and MTL, respectively. Each consecutive generation was referred to as F1, F2, and F3.

Briefly describe the acquisition of transfer host larvae (using the Chieh-qua population transfer host as an example), Cucumbers, loofahs, and bitter gourd, whose surfaces were sterilized with 75% alcohol, were placed in cages containing sexually mature adults of knucklehead populations for 24 h, respectively. These were used to obtain eggs of Chieh-qua populations, and the corresponding melons were used to rear them until the larvae reached maturity. Mature larvae were placed in the sterilized fine sand of suitable humidity to pupate. BTCF1, BTLF1, and BTMF1 adults were obtained after the pupae had fledged, and the adults were provided with the feed mentioned above and water separately for rearing. Subsequently, cucumbers, loofahs, and bitter gourds were placed in cages of BTCF1, BTLF1, and BTMF1 populations (surface sterilized) for adult oviposition. The larvae were reared using the corresponding melons and BTCF2, BTLF2, and BTMF2, and subsequent populations were obtained.

Each insect was disinfected with 75% ethanol for 1 min before dissection and washed thoroughly with ddH_2_O. In sterile PBS, sterilized forceps tear open the larval epidermis along the head, extract the intestinal tract, clean excess tissue, and retain the midgut. The entire dissection was performed on an ultra-clean bench sterilized in advance by UV light. All instruments, tools, and reagents used for dissection were sterilized or disinfected beforehand and placed on an ultra-clean bench to be sterilized by ultraviolet light irradiation 30 min before dissection. The last instar larvae were collected, and their intestines were dissected for each treatment. Each replicate contained the midgut of 15 larvae, and each treatment consisted of five replicates. The intestines were collected in 1.5 mL sterile centrifuge tubes, snap-frozen in liquid nitrogen, and stored at −80°C for subsequent use.

### 2.2 DNA extraction, PCR amplification, and sequencing

The gut of *B. cucurbitae* was well-ground in 1.5 mL sterile centrifuge tubes in liquid nitrogen using a manual homogenizer, and total DNA was extracted using the TIANamp Genomic DNA Kit (DP304; TIANGEN, Beijing, China). The V3–V4 variable region of bacterial 16S rDNA was PCR-amplified using specific primers (338F: 5′-ACTCCTACGGGAGGCAGCA-3′ and 806R: 5′-GGACTACNNGGGTATCTAAT-3′) and the amplification products used as templates to construct sequencing libraries (98°C for the 30s, followed by 25–27 cycles at 98°Cfor 15 s, 50°C for 30 s, and 72°C for 30 s and a final extension at 72°C for 5 min). After amplification, the PCR products of the same sample were mixed and detected by 2% agarose gel electrophoresis with a detection condition of 5 V/cm for 20 min. According to the manufacturer’s instructions, the extender was extracted and purified with the AxyPrep DNA gel extraction kit (Axygen Biosciences, Union City, CA, United States). Then, they were merged in equimolar quantities, and paired-end sequencing was performed on the NovaSeq-PE250 platform according to the standard protocol.

### 2.3 Bioinformatic and statistical analyses

Firstly, QIIME2 (2019.4) was used to excise the primer fragments of the sequences and discard the sequences that did not match the primers; then DADA2 was called via qiime dada2 denoise-paired for quality control, denoising, splicing, and de-chimerization, and clustered with 100% similarity to obtain ASVs (amplicon sequence variants). The QIIME2 classify-sklearn algorithm^1^ was used. For each feature sequence of ASVs, a pre-trained Naive Bayes classifier was used in the QIIME2 software with default parameters. The Greengenes database (Release 13.8, http://greengenes.secondgenome.com/) was selected for species annotation. The Chao1 index was used to assess alpha diversity and analyzed using SPSS 20.0 for multifactorial ANOVA. Tukey’s test was used for post-hoc comparisons. Taxonomic results, differences in species abundance, and principal coordinate analysis (PCoA) were analyzed using QIIME. Differences between groups were analyzed using 999 permutations to generate a permutation multivariate ANOVA. R software was used to perform Linear discriminant analysis (LDA, LDA > 2) Effect Size (LEfSe) analysis. We calculate the average abundance or overall quantity of the second-level pathways/classifications based on the chosen samples using a normalized path/group abundance table. Data were visualized using R software, Origin 2018, and GraphPad Prism 8.

### 2.4 Prediction of colony functional potential

First, the 16S rRNA gene sequences of known microbial genomes were aligned to construct an evolutionary tree and infer the gene function profiles of their common ancestors. The PICRUSt2 has completed this step. 16S rRNA signature sequences were aligned with the reference sequences, and a new evolutionary tree was constructed. Using the Castor hidden state prediction algorithm, the nearest sequence species of the feature sequences were inferred based on the gene family copy number corresponding to the reference sequences in the evolutionary tree, thus their gene family copy number. Note that when calculating the nearest sequence species index (NTSI) for each sequence, by default, if the sequence has an NTSI >2, it will be excluded from the subsequent analysis. The gene family copy number for each sample was calculated by combining the abundance of the characterized sequences for each sample. Note that here we used hierarchical processing, i.e., for each gene family of feature sequences, added the species information of the sequences and output the results in a hierarchical manner (i.e., the functional units of different feature sequences were not combined and processed), in order to realize the corresponding analysis of function and species. Finally, the gene families were “mapped” to various databases (KEGG database, MetaCyc data, and COG data), and the presence of metabolic pathways was inferred by default using MinPath to obtain the abundance data of metabolic pathways in each sample.

## 3 Results

### 3.1 Study on the gut microbes of *Bactrocera cucurbitae* transferred to different hosts

After removing singletons, we obtained 18,453,045 sequences from the gut microbes of the four groups of *B. cucurbitae* that fed on different hosts for three generations and generated 11,712 OTUs (Supplementary Table S1). A total of 180 samples identified 38 phyla, 92 classes, 229 orders, 401 families, 953 genus, and 1,298 species (Supplementary Table S2).

**Figure 1:**
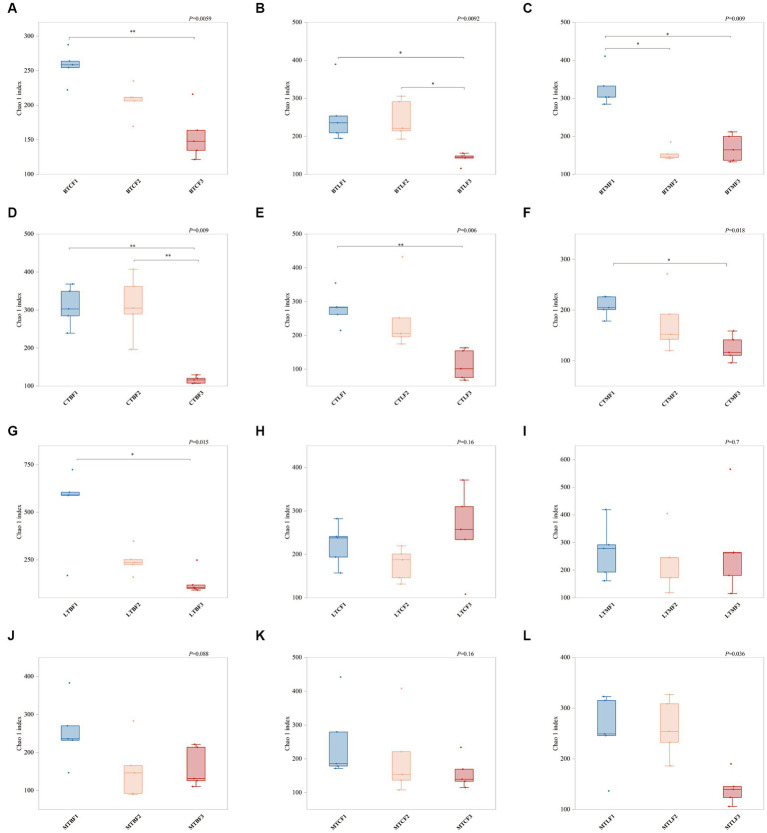
Changes in Chao1 index (±SE) after host shift of *Bactrocera cucurbitae.* * Denotes significant difference between the two groups at *p* = 0.05, ** denotes significant difference at *p* = 0.01. **(A–C)** Host shift of the Chieh-qua population to cucumber (BTC), loofah (BTL), and bitter melon (BTM). **(D–F)** Host shift of the cucumber population to Chieh-qua (CTB), loofah (CTL), and bitter melon (CTM). **(G–I)** Host shift of the loofah population to Chieh-qua (LTB), cucumber (LTC), and bitter melon (LTM). **(J–M)** Host shift of the bitter melon population to Chieh-qua (MTB), cucumber (MTC), and loofah (MTL). F1, F2, and F3 represent the first, second, and third generations after transfer, respectively.

The OTU numbers of different samples ranged from 115 to 633 (Supplementary Table S1). Comparison of the Chao1 coefficients for different populations transferred to the same host for different generations showed that most populations had a significant decrease after the third generation of transfer compared with those of the first generation (BTC, BTL, BTM, CTB, CTL, CTM, and LTB). BTM showed a significant decrease in Chao1 coefficients in the F2 generation and remained stable in the F3 generation, whereas those of BTL and CTM began to show a decrease only in the third generation (Figure 1). Chao1 coefficients of the gut microorganisms of *B. cucurbitae* transferred to the same host did not vary significantly between generations (Supplementary Figure S1), but ANOVA results showed that the Chao1 index was significantly influenced by the original host, host plant, and generation, and that there were significant interactions among these factors (Supplementary Table S3).

### 3.2 Effect of host shift on the gut microbes of the Chieh-qua population of *Bactrocera cucurbitae*

**Figure 2:**
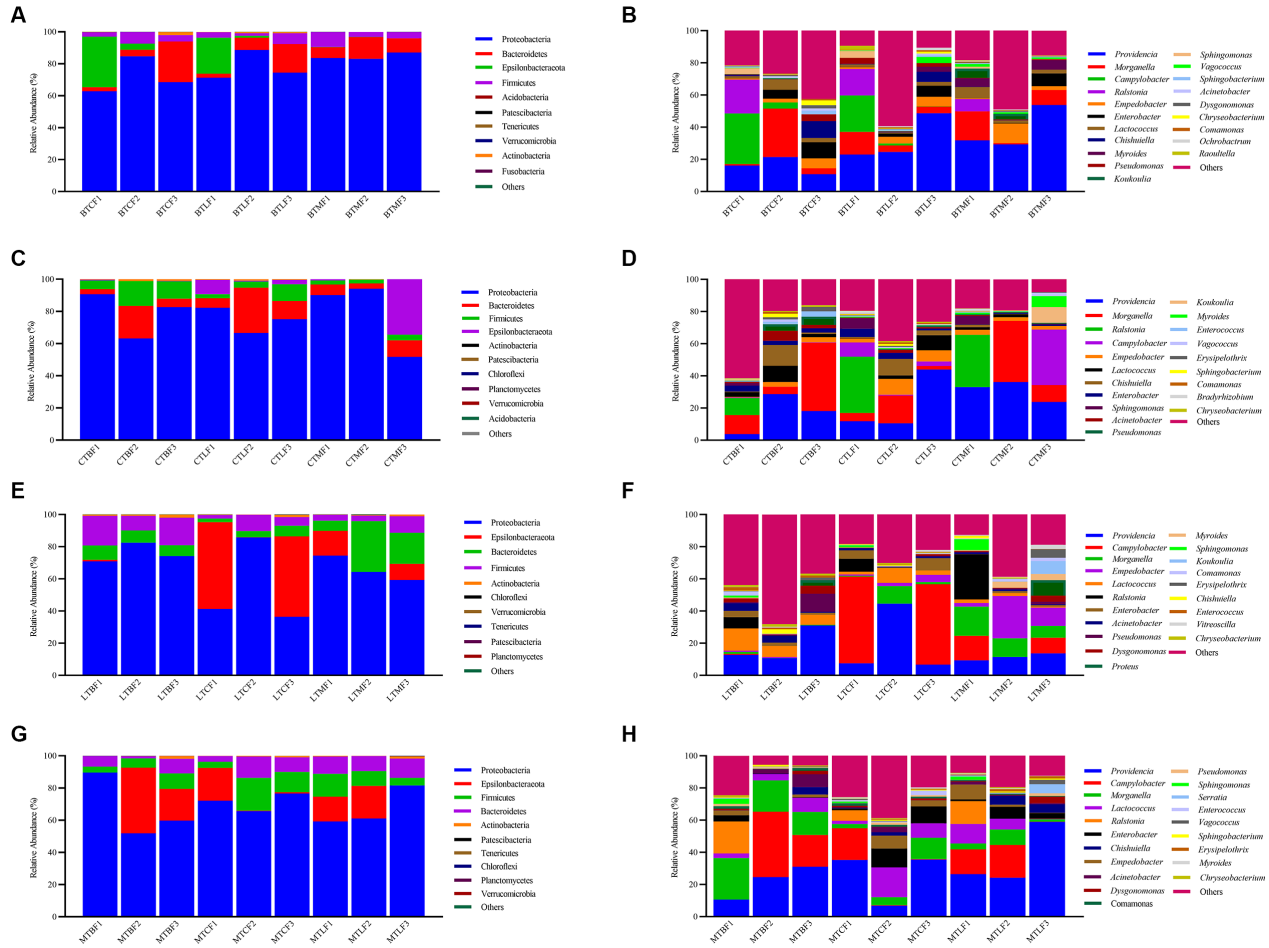
Relative abundance of the gut microbiota after host shift in *Bactrocera cucurbitae.* **(A,C,E,G)** Phylum and **(B,D,F,H)** genus levels. **(A,B)** Host shift of the Chieh-qua population to cucumber (BTC), loofah (BTL), and bitter melon (BTM). **(C,D)** Host shift of the cucumber population to Chieh-qua (CTB), loofah (CTL), and bitter melon (CTM). **(E,F)** Host shift of the loofah population to Chieh-qua (LTB), cucumber (LTC), and bitter melon (LTM). **(G,H)** Host shift of the bitter melon population to Chieh-qua (MTB), cucumber (MTC), and loofah (MTL). F1, F2, and F3 represent the first, second, and third generations after transfer, respectively.

After transferring to three other hosts, Proteobacteria (62.78–88.66%), Bacteroidetes (2.50–25.40%), Epsilonbacteraeota (0.01–31.56%), Firmicutes (1.60–9.36%), Actinobacteria (0.07–2.00%), Patescibacteria (0–0.06%), Tenericutes (0–0.02%), Verrucomicrobia (0–0.02%), Fusobacteria (0–0.03%), and Acidobacteria (0–0.01%) were the larval ten phylum with the highest percentage of intestinal bacteria. The relative abundance of Proteobacteria was the highest at the phylum level (62.78–88.66%), followed by that of Bacteroidetes (2.50–25.40%) and Epsilonbacteraeota (0.01–0.06%) (Supplementary Table S4). The relative abundance of Epsilonbacteraeota and Firmicutes was 0.10 to 31.57% and 1.60 to 9.36% in the first generation, respectively, and that of the four clades was 97.95 to 99.88% (Figure 2A). Epsilonbacteraeota was significantly reduced in F2 and F3. In contrast, Bacteroidetes accumulated significantly in F3 (Supplementary Table S4). At the genus level, *Providencia* (10.67–53.76%), *Morganella* (0.81–30.03%), *Campylobacter* (0.01–31.37%), *Ralstonia* (0–21.12%), *Empedobacter* (0.37–12.23%), *Enterobacter* (0.08–9.97%), *Lactococcus* (0.76–7.05%), *Chishuiella* (0.01–10.59%), *Myroides* (0–5.80%), *Pseudomonas* (0.07–4.27%) were the 10 strains with the highest percentage. The relative abundance of *Providencia* and *Morganella* ranged from 10.67 to 53.76% and 0.81 to 30.03%, respectively, and was higher in F3 than in F1 and F2 generations, except in the BTC group. Except for the BTC group, the relative abundance of *Morganella* in F3 ranged from 0.81 to 30.03% and was lower than that of F1 (Figure 2B). *Providencia’s* share of BTMF3 increases significantly. *Campylobacter* and *Ralstonia* concentrations showed a significant decrease in F3, particularly *Ralstonia,* which was mainly below the detection limit (Supplementary Table S5).

### 3.3 Effect of host shift on the gut microbes of the cucumber population of *Bactrocera cucurbitae*

After transferring to three other hosts, Proteobacteria (51.68–94.03%), Bacteroidetes (3.14–28.19%), Firmicutes (2.40–15.32%), Epsilonbacteraeota (0.01–34.46%), Actinobacteria (0.01 t-0.79%), Patescibacteria (0–0.19%), Chloroflexi (0–0.19%), Planctomycetes (0–0.04%), Verrucomicrobia (0–0.03%), Acidobacteria (0–0.02%) were the ten phyla with the highest percentage. The largest relative abundance of Proteobacteria (51.68–94.03%) was found in the cucumber populations after transfer to three additional hosts, followed by Bacteroidetes (3.14–28.19%), Epsilonbacteraeota (0.01–34.46%) (Supplementary Table S6). Firmicutes had a relative abundance of 2.40 to 15.32%, and these four clades were 98.82–99.98% (Figure 2C). Proteobacteria were markedly decreased in CTMF3, while Epsilonbacteraeota were notably augmented (Supplementary Table S6). At the genus level, *Providencia* (3.79–43.96%), *Morganella* (0.04–42.35%), *Ralstonia* (0–35.06%), *Campylobacter* (0–34.46%), *Empedobacter* (0.36–9.80%), *Lactococcus* (0.16–10.06%), *Chishuiella* (0–12.89%), *Enterobacter* (0.08–5.31%), *Sphingomonas* (0–6.55%), *Acinetobacter* (0.13–6.14%) were the top 10 genera of bacteria in the gut. The relative abundance of *Providencia* ranged from 3.79 to 43.96% and was lower in the CTM group at F3 than at F1 and higher in the CTB and CTL groups. However, the relative abundance of *Providencia* dropped in the CTB and CTM groups at F3 but increased in the CTL group. With a relative abundance ranging from 0.04 to 42.35%, *Morganella* in the F3 CTL group increased, whereas it declined in the CTB and CTM groups (Figure 2D). *Campylobacter* was significantly increased in CTMF3. *Ralstonia* was not detected in all F2 and F3 (Supplementary Table S7).

### 3.4 Effect of host shift on the gut microbes of the loofah population of *Bactrocera cucurbitae*

Proteobacteria (36.41–85.83%), Epsilonbacteraeota (0–53.95%), Bacteroidetes (2.15–31.54%), Firmicutes (2.31–18.53%), Actinobacteria (0.03–1.87%), Chloroflexi (0–0.16%), Verrucomicrobia (0–0.06%), Tenericutes (0–0.07%), Patescibacteria (0–0.05%), and Planctomycetes (0–0.04%) were the 10 highest percentage phyla of bacteria. The relative abundance of Proteobacteria was also highest after transferring to the other three hosts in the loofah population, ranging from 36.41 to 85.83%, followed by Bacteroidetes (2.15–31.54%) (Supplementary Table S8). The relative abundance of Epsilonbacteraeota in F3 was lower than that in F1, whereas that in LTBF3 and LTCF3 was lower than that in LTCF3. The relative abundance of Firmicutes was 2.31–17.16%, and that of these four clades was 98.05–99.74% (Figure 2E). Bacteroidetes were significantly increased in LTMF3 (Supplementary Table S8). At the genus level, the top 10 genus in the intestine were *Providencia* (6.74–44.64%), *Campylobacter* (0–53.93%), *Morganella* (0–18.11%), *Empedobacter* (0–26.22%), *Lactococcus* (0.62–13.86%), *Ralstonia* (0–28.09%), *Enterobacter* (0.04–7.72%), *Acinetobacter* (0.40–4.65%), *Pseudomonas* (0.03–11.24%), *Dysgonomonas* (0.01–5.04%). The relative abundance of *Providencia* at F3 was higher than that of F1 in both LTB and LTM groups, whereas it was lower at F3 than at F1 for the LTC group, with relative abundance ranging from 6.74 to 44.64%. *Morganella* behaved in the opposite way, with lower relative abundance in F3 than in F1 for the LTB and LTM groups and higher relative abundance in F3 than F1 in LTC, with relative abundances ranging from 0.02 to 18.12% (Figure 2F). Similarly, neither F2 nor F3 detected Ralstonia (Supplementary Table S9).

### 3.5 Effect of host shift on the gut microbes of the bitter gourd population of *Bactrocera cucurbitae*

After transfer of bitter melon populations to the remaining three hosts, the top 10 phyla were Proteobacteria (51.90–89.57%), Epsilonbacteraeota (0.04–40.69%), Firmicutes (3.68–20.495), Bacteroidetes (1.34–13.29%), Actinobacteria (0.06–1.64%), Patescibacteria (0–0.25%), Tenericutes (0–0.10%), Chloroflexi (0–0.07%), Planctomycetes (0–0.04%), Verrucomicrobia (0–0.05%) (Supplementary Table S10). The relative abundance of Proteobacteria ranged from 51.91 to 89.57%, Epsilonbacteraeota from 0.04 to 40.69%, Firmicutes from 3.67 to 20.49%, and Bacteroidetes from 1.34 to 13.28%, and the relative abundance of these four phyla was 98.23–99.82% (Figure 2G). Compared to F1, Epsilonbacteraeota was elevated in MTBF3 and decreased in MTCF3 and MTLF3, but the differences were significant (Supplementary Table S10). At the genus level, the top 10 genera in the larval gut were *Providencia* (6.79–58.99%), *Campylobacter* (0.02–40.68%), *Morganella* (1.51–25.81%), *Lactococcus* (0.30–18.60%), *Ralstonia* (0–19.94%), *Enterobacter* (0.02–11.71%), *Empedobacter* (0.01–9.17%), *Chishuiella* (0.03–5.52%), *Acinetobacter* (0.17–8.03%), *Dysgonomonas* (0.05–4.01%). The relative abundance of *Providencia* in the three transfer groups was higher in F3 than in F1, with relative abundances ranging from 6.79 to 58.99%. The relative abundance of *Morganella* in the MTC group was higher in F3 than in F1, whereas that in the MTB and MTL groups of F3 was lower than that in F1, with relative abundances ranging from 1.51 to 25.81% (Figure 2H). *Ralstonia* was also not detected in F2 and F3 (Supplementary Table S11).

### 3.6 Effects of original host, host plant, and generation on the gut microbes of *Bactrocera cucurbitae*

Three phyla were stable in all 12 groups of the transferred hosts: Proteobacteria, Bacteroidetes, and Firmicutes. At the genus level, two major genera, *Providencia* and *Morganella*, were stable in all samples, and the relative abundance ranged from 7.99 to 74.15%. In addition, *Lactococcus* was stable in all samples (Figure 2).

**Figure 3:**
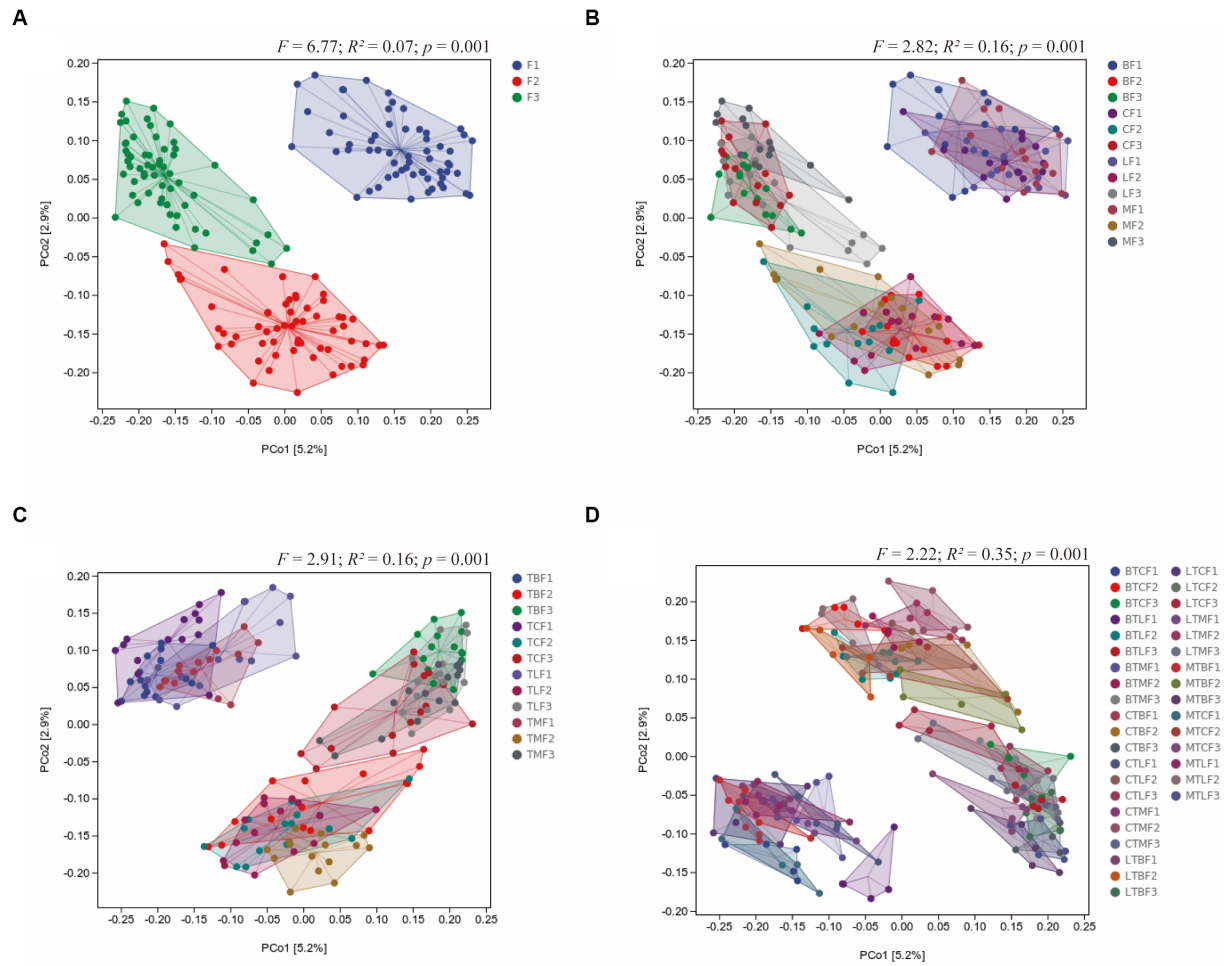
Principal coordinate analysis of the microbiota communities of *Bactrocera cucurbitae* after host shift based on Jaccard distance. Main group of **(A)** generations, **(B)** original host × generation, **(C)** host × generations, **(D)** original host × host × generation.

Principal coordinate analysis based on Jaccard distance was used to compare community similarity between samples with different grouping factors. The horizontal and vertical coordinates were the two most significant eigenvalues for sample differences, with contributions of 2.9 and 5.2% for the horizontal and vertical coordinates, respectively (Figure 3). Permutation multivariate ANOVA was used to analyze the effects and interactions of three different factors on gut microbes: *B. cucurbitae* original host (the host on which the *B. cucurbitae* feeds before transferring the host), generation (the generations that the *B. cucurbitae* has bred after transferring), and host (the host on which the *B. cucurbitae* feeds after transferring). The results showed that generations after the transfer had a significant effect on the gut microbes of *B. cucurbitae* (*R*^2^ = 0.07, *p* = 0.01) (Figure 3A), as did the original host × generations (*R*^2^ = 0.16, *p* = 0.001) (Figure 3B), generations × host (*R*^2^ = 0.16, *p* = 0.001) (Figure 3C), and original host × host × generation (*R*^2^ = 0.35, *p* = 0.001) (Figure 3D). Permutation multivariate ANOVA of the original host, host, and original host × host showed a *p* = 0.001, but the PCoA results showed that the differences between these groups were not significant (Supplementary Figure S2).

The Venn diagram shows that the gut microbes of *B. cucurbitae* overlapped after transfer from the same original host to different hosts, and the overlap of its gut microorganisms after rearing on the three hosts decreased gradually with the number of generations after transfer. When the Chieh-qua population was transferred to the other three hosts, the number of OTUs that overlapped for three consecutive generations was 135, 95, and 64, accounting for 6.67, 7.50, and 5.36% of the total, respectively. When the cucumber population was transferred to the other three hosts, the number of OTUs that overlapped for three consecutive generations was 119, 85, and 34, accounting for 6.87, 4.91, and 3.60% of the total, respectively; When the loofah population was transferred to the other three hosts, the number of OTUs that overlapped for three consecutive generations was 159, 90, and 99, accounting for 7.40, 5.79, and 5.86% of the total, respectively. When balsam pear was transferred to the other three hosts, 10.71, 6.20, and 3.55% of the total number of OTUs overlapped (Supplementary Figure S5).

**Figure 4:**
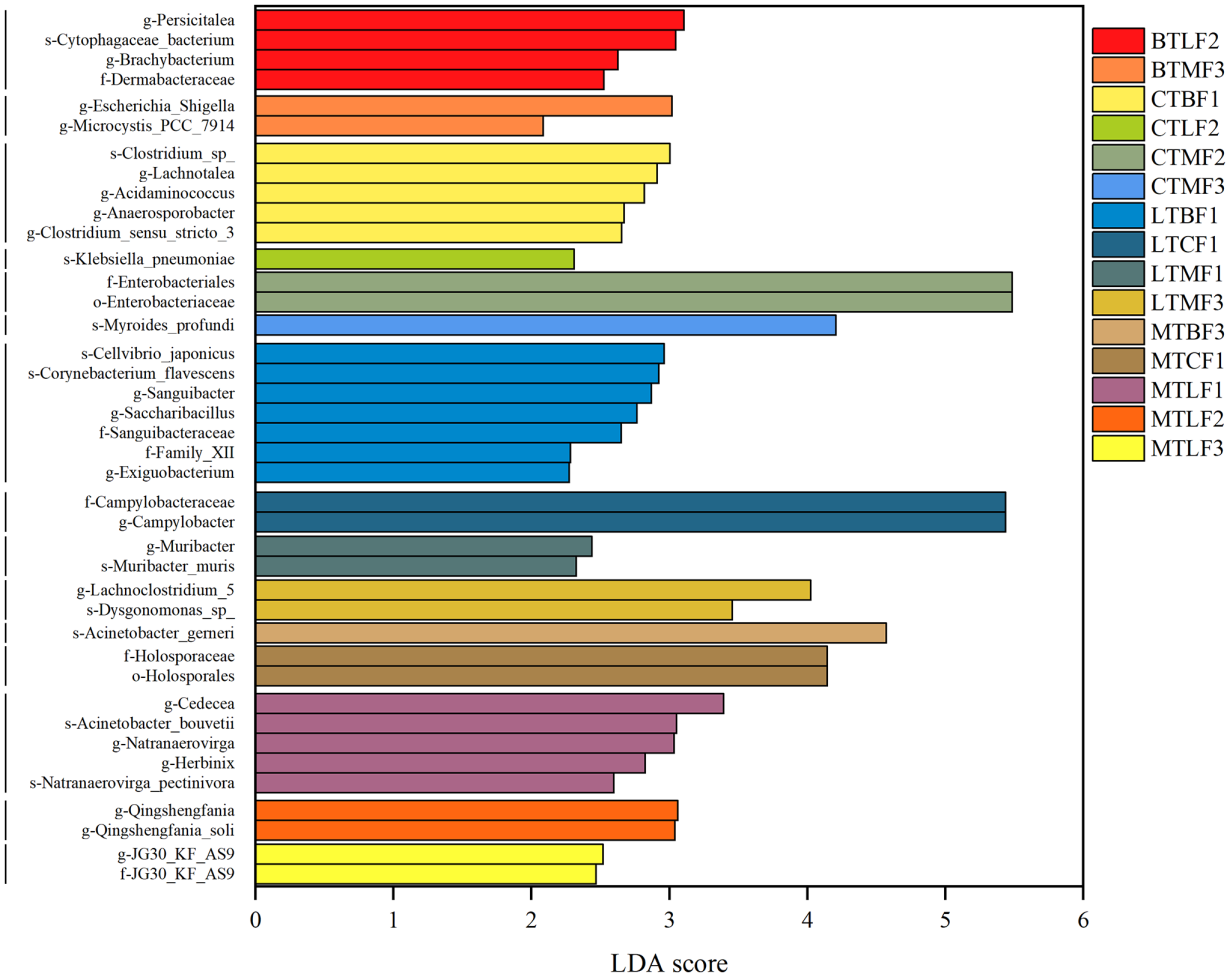
Bacterial taxa with LDA scores of host shift of the *Bactrocera cucurbitae*, from the phylum to genus levels, with an LDA score > 2. Columns of different colors represent host shift of the Chieh-qua population to loofah (BTL), and bitter melon (BTM); host shift of the cucumber population to Chieh-qua (CTB), loofah (CTL), and bitter melon (CTM); host shift of the loofah population to Chieh-qua (LTB), cucumber (LTC), and bitter melon (LTM); host shift of the bitter melon population to Chieh-qua (MTB), cucumber (MTC), and loofah (MTL). F1, F2, and F3 represent the first, second, and third generations after transfer, respectively.

To identify biomarkers with significant differences after the transfer of *B. cucurbitae* to different hosts, different taxa at different levels were examined using LEfSe with LDA >2 as a criterion. Among the Chieh-qua populations of *B. cucurbitae* that were transferred to different hosts, there were four differential groups in BTLF2, namely, *Persicitalea*, *Cytophagaceae_bacterium, Brachybacterium* and Dermabacteraceae. There were two taxa in BTMF3, *Escherichia Shigella* and *Microcystis PCC 7914* (Figure 4). Five taxa were present in CTBF1; the most significantly different taxon was *Clostridium* sp. the one in CTLF2 belonged to *Klebsiella pneumoniae*; the two in CTMF2 belonged to *Enterobacteriales*; and the *Myroides profundi* is the most significantly different in CTMF3 (Figure 4). Of the seven taxa in LTBF1, the most significantly different was *Cellvibrio japonicus;* the two taxa in LTCF1 belonged to Campylobacteraceae; of the two taxa in LTMF1, *Muribacter* was the most significantly different; and of the two taxa in LTMF3, the most significantly different was *Lachnoclostridium-5* (Figure 4). There was one taxon in MTBF3 belonging to *Acinetobacter gerneri*; MTCF1 contained two belonging to Holosporales; of the five in MTLF1, *Cedecea* was the most significantly different; of the two in MTLF2, belong to *Qingshengfania*; and MTLF3 had two belonging to JG30-KF-AS9 (Figure 4). Through LEfSe (LDA >4) to find biomarkers that differed significantly among samples fed with different host plants, five taxa belonging to Firmicutes and *Pseudomonadales* were found in the gut of *B. cucurbitae* larvae reared on Chieh-qua; one belonging to *Chishuiella* in those reared on cucumber; one belonging to *Enterobacter*; one belonging to *Chishuiella* in those reared on loofah; and six belonging to *Empedobacter*, *Flavobacteriaceae*, and Cardiobacteriales in those reared on bitter gourd (Supplementary Figure S3).

### 3.7 Functional analysis of the *Bactrocera cucurbitae* larvae’s gut microbes

The function of *B. cucurbitae* gut microbes was predicted using PICRUSt2, and the abundance values of metabolic pathways were determined using R software. Heat maps were also used to visualize the function of *B. cucurbitae* gut microbes transferred to different hosts from different original hosts. The total statistical map showed that the metabolic functions of *B. cucurbitae* intestinal bacteria were clustered into Biosynthesis, Degradation/Utilization/Assimilation, Detoxification, and Generation of Precursor Metabolite and Energy. The highest relative abundance measured was for Biosynthesis (68.72%), followed by Degradation/Utilization/Assimilation (15.66%), Generation of Precursor Metabolite and Energy (11.54%), and Metabolic Clusters (2.26%), Macromolecule Modification (0.80%), Glycan Pathways (0.57%), and Detoxification (0.43%) (Supplementary Figure S4). In Biosynthesis the highest abundances were for Cofactor, Prosthetic Group, Electron Carrier, and Vitamin Biosynthesis (17.57%), Amino Acid Biosynthesis (14.55%), Nucleoside and Nucleotide Biosynthesis (12.67%), and Fatty Acid and Lipid Biosynthesis (10.43%) (Supplementary Figure S4). In Degradation/Utilization/Assimilation the carbohydrate degradation, carboxylate degradation, nucleoside and nucleotide degradation, and secondary metabolite degradation were the five most enriched pathways (Supplementary Figure S4).

**Figure 5:**
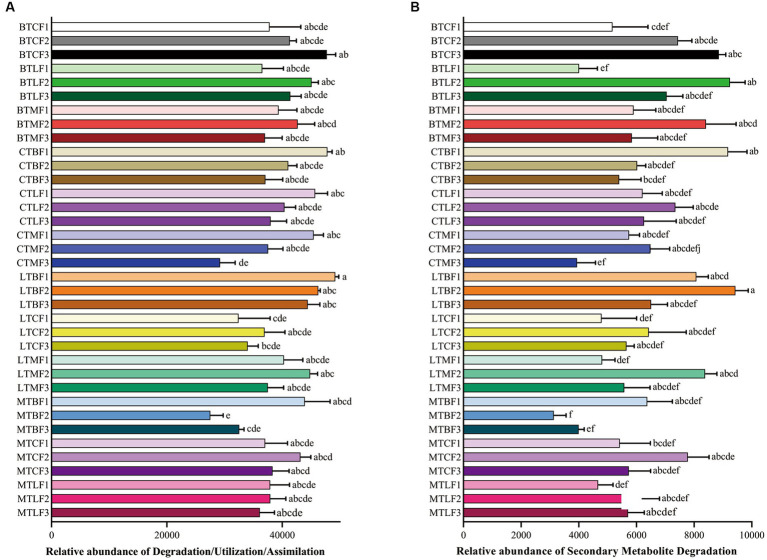
Comparison of predicted GO functions of the gut bacteria of *Bactrocera cucurbitae* after host shift. Data represented by columns bearing the different letters were significantly different (Tukey, *p* = 0.05). **(A)** Relatiave abundance (±SE) of Degradation/Utilization/Assimilation; **(B)** Relatiave abundance (±SE) of Secondary Metabolite Degradation. Each column represents host shift of the Chieh-qua population to cucumber (BTC), loofah (BTL), and bitter melon (BTM); host shift of the cucumber population to Chieh-qua (CTB), loofah (CTL), and bitter melon (CTM); host shift of the loofah population to Chieh-qua (LTB), cucumber (LTC), and bitter melon (LTM); host shift of the bitter melon population to Chieh-qua (MTB), cucumber (MTC), and loofah (MTL). F1, F2, and F3 represent the first, second, and third generations after transfer, respectively.

Secondary Metabolite Degradation was more abundant in BTC, BTL, LTC, LTM, MTC and MTL in the F3 groups than in the F1 groups and lower in CTB, CTM, LTB and MTB in the F3 groups than in the F1 groups, although the difference is not significant (Figure 5).

## 4 Discussion

Throughout all phases of host life, the gut bacteria of insects perform crucial functions (Sharon et al., 2010; Storelli et al., 2011, 2018; Clark et al., 2015). Interactions between animals and microorganisms might vary slightly depending on the host’s symbiotic bacteria and their developmental phases (Turnbaugh et al., 2007; Chandler et al., 2011). Food has a significant impact on essential traits of the host and greatly influences the makeup of symbiotic bacteria in the insect’s stomach (Chandler et al., 2011; Jones et al., 2019; Mason et al., 2021). Insect behavior may change as a result of altered gut microorganisms brought on by diet (Leite-Mondin et al., 2021). Numerous studies have demonstrated that insect gut bacteria play a critical role in improving insect host and environmental adaptations, as well as in helping insects metabolize harmful substances ingested into the body, such as pesticides, external pathogens, and secondary metabolites from plants (Motta et al., 2018; Zhang et al., 2020). To understand how gut bacteria aid in the adaptation of insects to their hosts, it is crucial to study the changes in gut microbial composition that occur when insects are transferred to other hosts. To provide a theoretical foundation for understanding the dynamics of gut bacteria in *B. cucurbitae* during host adaptation, we analyzed the changes in gut bacterial communities in three consecutive generations that were transferred to different hosts.

Different diets affect the composition of insect gut microbes and insect behavior (Wong et al., 2017; Paniagua Voirol et al., 2018; Ge et al., 2021). Changes in the host usually cause drastic changes in insect gut microbes (Yang et al., 2020). Gut microbes can respond to external stimuli faster than insects because of their faster evolution rate (Rosenberg and Zilber-Rosenberg, 2018). Different *B. cucurbitae* populations were transferred to three different hosts, where a significant decline in gut microbial diversity was observed in the second and third generations. This finding suggests that the gut microbiota of *B. cucurbitae* can undergo rapid changes within 2–3 generations after host replacement, which may help *B. cucurbitae* adapt to their hosts. Insect gut microorganisms are influenced by the environment, host plants, and current generations, and there are some interactions between these variables (Jones et al., 2019; Yang et al., 2022). The interaction between insect lineages and hosts can lead to changes in the gut microbiota of insects, which may lead to adaptive changes in insects (Jose et al., 2023). The original host, generations, and hosts after transfer were all found to significantly interact with the gut microbes of *B. cucurbitae* in the current study, suggesting that they might affect the colonization of gut microbes.

Many core gut microbes are present throughout the host’s life stages and respond to external factors to form a unique homeostasis in the gut (Hana et al., 2023). In this study, Proteobacteria, Bacteroidetes, Epsilonbacteraeota, and Firmicutes were the dominant phyla in the gut of *B. cucurbitae* after the transfer of different populations to different hosts. Proteobacteria, Bacteroidetes, and Firmicutes were also prevalent in the guts of *Bactrocera* species and *B. cucurbitae* in previous studies (Hadapad et al., 2019; Asimakis et al., 2021; Choudhary et al., 2021). The number of OTUs coexisting in the three host populations gradually decreased when the same population of *B. cucurbitae* was moved to three different hosts for feeding. This suggests that, while the core flora does not entirely vanish, differences in the gut microbes of *B. cucurbitae* gradually grow as the number of generations living on different hosts increases. The ability of insects to adapt to host plants may be enhanced by the stable presence of microbes in their stomachs (Yang et al., 2020). The composition of insect gut microbes is also associated with secondary metabolites in host plants and influences insect adaptation to host plants (Zhang et al., 2020). Abundance of the Secondary Metabolite Degradation pathway increased when nodule populations were transferred to live on cucumber, loofah, and bitter melon for three generations, whereas this decreased when cucumber, loofah, and bitter melon populations were transferred to live on nodules for three generations. This may be related to the presence of secondary metabolites in cucumber, loofah, and bitter melon. Cucurbitaceae crops can usually synthesize bitter triterpenoids, such as cucurbitins, which have certain anti-insect effects (Balkema-Boomstra et al., 2003; Shah et al., 2014). The cucurbitane triterpenoids characteristic of bitter melon are classified as a special cucurbitin, which has a unique structure (Chen et al., 2005). Therefore, the different secondary metabolites contained in melon crops may also be one of the reasons for the changes in the gut microbes of *B. cucurbitae*. Predominant intestinal bacteria in both first-instar larvae and adult females of the cucumber-feeding *B. cucurbitae* are associated with digestive functions (Choudhary et al., 2021). Proteobacteria and Firmicutes are dominant in the gut of many insects and may be associated with amino acid and carbohydrate metabolism and membrane transport pathways in the host. *Providencia* and *Morganella* are widespread in the gut of *Bactrocera* sp. (Gujjar et al., 2017; Rashid et al., 2018; Zhang et al., 2021). Two genera of Enterobacteriaceae, *Providencia* and *Morganella*, were stably present in different host populations with high relative abundances. In contrast, the remaining genera were not stably present across samples, indicating that *Providencia* and *Morganella* are the core groups of bacteria in the gut of *B. cucurbitae* larvae, although their relative abundance fluctuated with the host. Enterobacteriaceae is an important group of bacteria in many insects and is involved in many important life activities (Wang et al., 2014; Chen et al., 2021). *Providencia* significantly shortens larval development time and increases pupal weight in *B. dorsalis*, whereas in houseflies (*Musca domestica*), it inhibits the growth of beneficial bacteria and reduces larval humoral immunity (Gichuhi et al., 2020; Zhang Q. et al., 2022). *Morganella* usually negatively affects insects; however, in *B. dorsalis,* it is involved in nitrogen metabolism (Zhang et al., 2021; Ren et al., 2022). Therefore, further experiments are required to investigate the specific functions of *Providencia* and *Morganella* in the gut of *B. cucurbitae*. Many symbiotic bacteria in insects can vertically spread between parents and offspring, and these symbiotic bacteria play an important role in insect life activities (Guo et al., 2017). Therefore, studying the differences in gut microbiota between parents and offspring is of great significance, and further research is needed in subsequent studies to investigate the differences in gut microbiota between the original host and the host shift in *B. cucurbitae*. Gut microorganisms are altered during the process of host shift in insects, and at the same time, the survival and development of insects are also affected by this process (Yang et al., 2020). In this study, the changes in gut microorganisms during host shift were characterized and will be further investigated in future studies in the context of survival and developmental changes in *B. cucurbitae*.

## 5 Conclusion

In summary, the results of this study show that the gut microbial alpha diversity of *B. cucurbitae* feeding on melon was significantly influenced by the original host × host × generations, and the analysis of differences between groups showed the same results. *Providencia* and *Morganella*, gut bacteria that are stably present and in high abundance, play an important role during *B. cucurbitae* host transfer. Functional prediction analysis showed that the abundance of secondary metabolite degradation by *B. cucurbitae* gut microbes was reduced in flies grown on Chieh-qua, suggesting that the gut microbes may be associated with the host’s secondary metabolite pathway. However, the important functions of *Providencia* and *Morganella* in the gut of *B. cucurbitae*, in which secondary metabolites play a role in the host plant, need to be verified through further experiments. The results of these experiments may provide a theoretical basis for the study of gut microbes.

## Data availability statement

The datasets presented in this study can be found in online repositories. The names of the repository/repositories and accession number(s) can be found at: NCBI – PRJNA999388.

## Ethics statement

The manuscript presents research on animals that do not require ethical approval for their study.

## Author contributions

ZT: Conceptualization, Investigation, Writing – original draft. LC: Data curation, Visualization, Writing – review & editing. GC: Methodology, Writing – review & editing. JW: Project administration, Writing – review & editing. CM: Project administration, Writing – review & editing. YZ: Project administration, Writing – review & editing. XG: Supervision, Writing – review & editing. HC: Supervision, Writing – review & editing. ZZ: Conceptualization, Writing – review & editing.

## Footnotes

^1^https://github.com/QIIME2/q2-feature-classifier

## Supplementary material

Supplementary Table S1 Total sequencing reads and OTU statistics in five repetitions of *Bactrocera cucurbitae* 36 samples.

Supplementary Table S2 Number of identified gut microorganism taxonomic categories after host shift of *B. cucurbitae*.

Supplementary Table S3 ANOVA of Alpha diversity indices of gut microorganism communities in the hosts shift of *B. cucurbitae*.

Supplementary Table S4 Effect of Host Shift on the top 5 Gut Microbes of the Chieh-qua Population of *B. cucurbitae* at the phylum level.

Supplementary Table S5 Effect of Host Shift on the top 5 Gut Microbes of the Chieh-qua Population of *B. cucurbitae* at the genus level.

Supplementary Table S6 Effect of Host Shift on the top 5 Gut Microbes of the Cucumber Population of *B. cucurbitae* at the phylum level.

Supplementary Table S7 Effect of Host Shift on the top 5 Gut Microbes of the Cucumber Population of *B. cucurbitae* at the genus level.

Supplementary Table S8 Effect of Host Shift on the top 5 Gut Microbes of the Loofah Population of *B. cucurbitae* at the phylum level.

Supplementary Table S9 Effect of Host Shift on the top 5 Gut Microbes of the Loofah Population of *B. cucurbitae* at the genus level.

Supplementary Table S10 Effect of Host Shift on the top 5 Gut Microbes of the Bitter Gourd Population of *B. cucurbitae* at the phylum level.

Supplementary Table S11 Effect of Host Shift on the top 5 Gut Microbes of the Bitter Gourd Population of *B. cucurbitae* at the genus level.

Supplementary Figure S1 Changes in Chao1 index (±SE) after host shift of *Bactrocera cucurbitae*.

Supplementary Figure S2 Principal coordinate analysis of the microbiota communities of *Bactrocera cucurbitae* after host shift based on Jaccard distance.

Supplementary Figure S3 Bacterial taxa with LDA scores of host shift of the *Bactrocera cucurbitae*, from the phylum to genus levels, with an LDA score >2.

Supplementary Figure S4 Comparison of predicted GO functions of the gut bacteria of *Bactrocera cucurbitae* after host shift.

Supplementary Figure S5 Venn diagram of microbiota community OTUs of *Bactrocera cucurbitae* after host shift.

Supplementary Figure S6 Comparison of predicted GO functions of the gut bacteria of *Bactrocera cucurbitae* after host shift.
