# Supplementary material for: Effect of host shift on the gut microbes of Bactrocera cucurbitae (Coquillett) (Diptera: Tephritidae)
Source: Front Microbiol. 2023 Nov 21;14:1264788. doi: 10.3389/fmicb.2023.1264788 (PMC10703373; doi:10.3389/fmicb.2023.1264788)
Supplement: Supplementary Table S1 — Total sequencing reads and OTU statistics in five repetitions of Bactrocera cucurbitae 36 samples. [file Table_1.DOCX]

**Supplementary Table 1** Total sequencing reads and OTU statistics in five repetitions of *Bactrocera cucurbitae* 36 samples.

| Treatment | Input | Non#singleton | Number of OTUs |
| --- | --- | --- | --- |
| BTCF1 | 134865.6±16414.8 | 104589.4±15581.1 | 249 |
| BTCF2 | 135236.4±891.5 | 113192.6±5091.4 | 150 |
| BTCF3 | 137388.6±6062.7 | 112198.8±8057.6 | 201 |
| BTLF1 | 113972.0±9911.9 | 101748.2±6526.5 | 406 |
| BTLF2 | 138370.8±8226.7 | 125663.2±12899.4 | 229 |
| BTLF3 | 141530.8±4601.6 | 128592.8±4253.7 | 200 |
| BTMF1 | 89240.6±4812.7 | 71162.6±5538.2 | 469 |
| BTMF2 | 81701.8±3666.7 | 70385.0±8323.7 | 115 |
| BTMF3 | 96638±6836.7 | 88397.6±8063.4 | 236 |
| CTBF1 | 133689.6±19223.8 | 113256.8±17608.1 | 261 |
| CTBF2 | 142278.8±1632.2 | 121974.2±1638.2 | 403 |
| CTBF3 | 140349.8±5730.9 | 119562.4±8921.2 | 155 |
| CTLF1 | 127254.2±11728.4 | 108182.8±10415.9 | 379 |
| CTLF2 | 141355.4±5512.8 | 129715.6±4760.7 | 310 |
| CTLF3 | 126463.4±7398.3 | 109591.4±13255.2 | 160 |
| CTMF1 | 95347.4±1535.5 | 85723.2±2635.5 | 170 |
| CTMF2 | 78757.4±2570.9 | 72589.6±2312.6 | 125 |
| CTMF3 | 87702.0±25682.9 | 79121.2±26393.3 | 174 |
| LTBF1 | 76497.8±3354.0 | 71131.8±2471.8 | 633 |
| LTBF2 | 80169.0±8021.5 | 67079.6±13124.9 | 228 |
| LTBF3 | 85615.8±7340.8 | 77486.6±8542.8 | 209 |
| LTCF1 | 142364.8±5600.8 | 120871.2±12757.0 | 122 |
| LTCF2 | 143038.2±5011.7 | 122935.8±5617.0 | 158 |
| LTCF3 | 140344.8±3548.6 | 114278±6121.0 | 291 |
| LTMF1 | 143600.6±2920.5 | 130731.2±6513.7 | 354 |
| LTMF2 | 121253.6±16024.5 | 104226.4±17358.2 | 308 |
| LTMF3 | 131628.0±11504.1 | 117037±17087.7 | 460 |
| MTBF1 | 140620.2±1850.6 | 117331.4±1924.3 | 206 |
| MTBF2 | 141590.6±5941.8 | 110114±11109.5 | 162 |
| MTBF3 | 137693.0±3493.1 | 114434.6±5539.4 | 248 |
| MTCF1 | 84818.8±8582.9 | 75526±8268.7 | 217 |
| MTCF2 | 76968.2±6287.2 | 64976.2±9947.5 | 267 |
| MTCF3 | 87177.4±10040.8 | 78684.2±12018.0 | 246 |
| MTLF1 | 130249.8±17726.4 | 116563.8±20225.6 | 220 |
| MTLF2 | 138261.8±3786.5 | 125193.2±5339.5 | 257 |
| MTLF3 | 117020.8±6381.0 | 106360.6±8262.1 | 214 |
| Total | 21305279 | 18453045 | 11712 |

**Supplementary Table 2** Number of identified gut microorganism taxonomic categories after host shift of *B. cucurbitae*

| Treatment | Phylum | Class | Order | Family | Genus | Species |
| --- | --- | --- | --- | --- | --- | --- |
| BTLF3 | 12 | 20 | 44 | 75 | 149 | 183 |
| BTCF1 | 14 | 22 | 59 | 96 | 168 | 207 |
| BTCF2 | 12 | 21 | 43 | 75 | 156 | 195 |
| BTLF1 | 20 | 37 | 83 | 137 | 251 | 280 |
| BTLF2 | 17 | 29 | 56 | 94 | 177 | 230 |
| BTMF1 | 11 | 20 | 49 | 81 | 166 | 206 |
| BTMF2 | 14 | 21 | 40 | 66 | 123 | 149 |
| BTMF3 | 20 | 31 | 62 | 98 | 173 | 210 |
| BTCF3 | 15 | 23 | 45 | 78 | 169 | 209 |
| CTBF1 | 14 | 22 | 58 | 102 | 197 | 248 |
| CTBF2 | 24 | 59 | 119 | 184 | 296 | 291 |
| CTBF3 | 13 | 23 | 39 | 76 | 145 | 161 |
| CTLF1 | 10 | 17 | 50 | 83 | 169 | 207 |
| CTLF2 | 19 | 36 | 69 | 100 | 198 | 235 |
| CTLF3 | 11 | 20 | 41 | 68 | 120 | 128 |
| CTMF1 | 15 | 29 | 58 | 38 | 86 | 147 |
| CTMF2 | 23 | 46 | 88 | 125 | 200 | 185 |
| CTMF3 | 10 | 16 | 35 | 67 | 129 | 156 |
| LTBF1 | 15 | 24 | 58 | 104 | 188 | 274 |
| LTBF2 | 11 | 21 | 47 | 73 | 125 | 170 |
| LTBF3 | 13 | 21 | 42 | 73 | 170 | 204 |
| LTCF1 | 11 | 18 | 40 | 38 | 67 | 129 |
| LTCF2 | 12 | 23 | 44 | 77 | 154 | 178 |
| LTCF3 | 10 | 18 | 45 | 86 | 191 | 249 |
| LTMF1 | 11 | 20 | 45 | 71 | 145 | 175 |
| LTMF2 | 25 | 56 | 108 | 156 | 260 | 250 |
| LTMF3 | 14 | 25 | 52 | 87 | 193 | 258 |
| MTBF1 | 14 | 24 | 52 | 85 | 157 | 193 |
| MTBF2 | 19 | 38 | 78 | 114 | 150 | 145 |
| MTBF3 | 13 | 22 | 48 | 83 | 151 | 194 |
| MTCF1 | 13 | 25 | 58 | 88 | 154 | 197 |
| MTCF2 | 14 | 27 | 50 | 80 | 156 | 200 |
| MTCF3 | 14 | 22 | 44 | 79 | 140 | 178 |
| MTLF1 | 14 | 25 | 51 | 85 | 175 | 224 |
| MTLF2 | 14 | 25 | 42 | 68 | 119 | 168 |
| MTLF3 | 14 | 29 | 59 | 98 | 157 | 170 |
| Total | 38 | 92 | 229 | 401 | 953 | 1,298 |

**Supplementary Table 3** ANOVA of Alpha diversity indices of gut microorganism communities in the hosts shift of *B. cucurbitae*.

| Factor | F | Sig. |
| --- | --- | --- |
| Original | 7.81 | .000 |
| Host | 4.98 | .003 |
| Generation | 69.06 | .000 |
| Original * Host | 3.23 | .009 |
| Original * Generation | 3.57 | .003 |
| Host * Generation | 8.87 | .000 |
| Original * Host * Generation | 9.26 | .000 |

**Supplementary table 4** Effect of Host Shift on the top 5 Gut Microbes of the Chieh-qua Population of *B. cucurbitae* at the phylum level

| Treatment | Proteobacteria(%) | Epsilonbacteraeota(%) | Bacteroidetes(%) | Firmicutes(%) | Actinobacteria(%) |
| --- | --- | --- | --- | --- | --- |
| BTCF1 | 62.78±13.80 a | 31.57±15.03 a | 2.58±0.83 b | 2.85±0.80 a | 0.16±0.05 b |
| BTLF1 | 71.27±10.33 a | 22.60±9.40 ab | 2.50±1.42 b | 3.34±1.11 a | 0.18±0.05 b |
| BTMF1 | 83.65±5.82 a | 0.13±0.05 b | 6.73±2.73 b | 9.36±3.70 a | 0.09±0.06 b |
| BTCF2 | 84.69±3.69 a | 3.88±2.62 ab | 4.03±1.11 b | 7.27±4.28 a | 0.11±0.02 b |
| BTLF2 | 88.66±2.33 a | 1.21±0.85 b | 7.63±1.45 b | 1.60±0.34 a | 0.80±0.12 ab |
| BTMF2 | 83.13±5.97 a | 0.10±0.03 b | 13.58±5.44 ab | 3.06±1.12 a | 0.10±0.05 b |
| BTCF3 | 68.47±3.07 a | 0.06±0.04 b | 25.40±2.43 a | 4.03±2.14 a | 2.00±0.51 a |
| BTLF3 | 74.43±7.30 a | 0.05±0.01 b | 17.84±7.84 ab | 6.68±2.70 a | 0.98±0.95 ab |
| BTMF3 | 87.08±3.44 a | 0.01±0.00 b | 8.89±1.63 ab | 3.91±3.00 a | 0.07±0.02 b |

a) Mean±SE. Means within the same column followed by different letters are significantly different at *P* ≤ 0.05, as determined by an ANOVA: Tukey test.

**Supplementary table 5** Effect of Host Shift on the top 5 Gut Microbes of the Chieh-qua Population of *B. cucurbitae* at the genus level

| Treatment | *Providencia*(%) | *Morganella*(%) | *Campylobacter*(%) | *Ralstonia*(%) | *Lactococcus*(%) |
| --- | --- | --- | --- | --- | --- |
| BTCF1 | 16.09±3.16 ab | 0.96±0.41 b | 31.37±15.09 a | 21.12±11.71 a | 1.27±0.44 a |
| BTLF1 | 22.93±4.57 ab | 14.19±8.16 ab | 22.58±9.39 ab | 16.50±4.76 ab | 1.61±0.73 a |
| BTMF1 | 31.83±12.31 ab | 17.99±11.05 ab | 0.06±0.03 b | 7.59±3.44 ab | 7.05±3.17 a |
| BTCF2 | 21.48±7.43 ab | 30.03±6.34 a | 3.87±2.61 ab | 0 b | 6.65±4.17 a |
| BTLF2 | 24.60±9.88 ab | 4.03±2.49 b | 1.21±0.85 b | 0 b | 0.77±0.18 a |
| BTMF2 | 29.32±10.23 ab | 0.81±0.35 b | 0.03±0.02 b | 0 b | 1.48±0.63 a |
| BTCF3 | 10.67±2.94 b | 3.70±2.68 b | 0.05±0.03 b | 0 b | 2.58±2.23 a |
| BTLF3 | 48.70±10.61 ab | 4.12±1.17 b | 0.05±0.01 b | 0 b | 2.36±0.91 a |
| BTMF3 | 53.76±11.93 a | 9.35±4.27 ab | 0.01±0.00 b | 0 b | 2.33±2.04 a |

a) Mean±SE. Means within the same column followed by different letters are significantly different at *P* ≤ 0.05, as determined by an ANOVA: Tukey test.

**Supplementary table 6** Effect of Host Shift on the top 5 Gut Microbes of the Cucumber Population of *B. cucurbitae* at the phylum level

| Treatment | Proteobacteria(%) | Epsilonbacteraeota(%) | Bacteroidetes(%) | Firmicutes(%) | Actinobacteria(%) |
| --- | --- | --- | --- | --- | --- |
| CTBF1 | 90.65±2.50 ab | 0.45±0.16 b | 3.14±0.51 b | 5.46±1.92 a | 0.25±0.03 a |
| CTLF1 | 82.21±6.71 ab | 8.82±7.39 b | 5.90±1.37 b | 2.58±0.45 a | 0.35±0.15 a |
| CTMF1 | 90.16±1.86 ab | 0.72±0.14 b | 6.52±1.31 b | 2.51±0.50 a | 0.04±0.02 a |
| CTBF2 | 63.09±5.48 bc | 0.01±0.00 b | 20.41±6.08 ab | 15.32±3.56 a | 0.76±0.25 a |
| CTLF2 | 66.53±9.38 abc | 0.58±0.35 b | 28.19±9.00 a | 3.86±0.64 a | 0.78±0.32 a |
| CTMF2 | 94.03±1.97 a | 0.04±0.02 b | 3.34±1.59 b | 2.41±0.86 a | 0.08±0.06 a |
| CTBF3 | 82.63±5.81 ab | 0.31±0.30 b | 5.35±2.64 b | 10.70±3.39 a | 0.79±0.45 a |
| CTLF3 | 75.16±6.53 abc | 2.78±2.35 b | 11.29±4.31 ab | 10.38±6.93 a | 0.39±0.26 a |
| CTMF3 | 51.68±8.08 c | 34.46±11.33 a | 10.38±4.90 ab | 3.46±1.39 a | 0.01±0.01 a |

a) Mean±SE. Means within the same column followed by different letters are significantly different at *P* ≤ 0.05, as determined by an ANOVA: Tukey test.

**Supplementary table 7** Effect of Host Shift on the top 5 Gut Microbes of the Cucumber Population of *B. cucurbitae* at the genus level

| Treatment | *Providencia*(%) | *Morganella*(%) | *Campylobacter*(%) | *Ralstonia*(%) | *Lactococcus*(%) |
| --- | --- | --- | --- | --- | --- |
| CTBF1 | 3.79±0.86 c | 11.81±5.75 abc | 0.42±0.16 b | 10.52±1.83 b | 2.90±1.18 a |
| CTLF1 | 11.81±2.63 bc | 5.06±4.63 bc | 8.81±7.39 b | 35.06±8.33 a | 0.22±0.08 a |
| CTMF1 | 33.04±2.63 abc | 0.04±0.03 c | 0.09±0.06 b | 32.36±3.60 a | 1.69±0.34 a |
| CTBF2 | 28.62±4.67 abc | 4.69±1.22 bc | 0.00±0.00 b | 0 b | 10.06±3.12 a |
| CTLF2 | 10.57±6.54 bc | 17.14±11.00 abc | 0.58±0.35 b | 0 b | 2.16±0.53 a |
| CTMF2 | 36.13±10.78 ab | 38.02±11.54 ab | 0.04±0.02 b | 0 b | 1.73±0.69 a |
| CTBF3 | 18.15±6.47 abc | 42.35±9.95 a | 0.31±0.30 b | 0 b | 2.06±1.39 a |
| CTLF3 | 43.96±11.00 a | 2.19±1.20 c | 2.78±2.35 b | 0 b | 9.47±7.07 a |
| CTMF3 | 23.78±6.29 abc | 10.53±7.93 abc | 34.46±11.33 a | 0 b | 0.16±0.16 a |

a) Mean±SE. Means within the same column followed by different letters are significantly different at *P* ≤ 0.05, as determined by an ANOVA: Tukey test.

**Supplementary table 8** Effect of Host Shift on the top 5 Gut Microbes of the Loofah Population of *B. cucurbitae* at the phylum level

| Treatment | Proteobacteria(%) | Epsilonbacteraeota(%) | Bacteroidetes(%) | Firmicutes(%) | Actinobacteria(%) |
| --- | --- | --- | --- | --- | --- |
| LTBF1 | 70.99±5.69 ab | 0.91±0.44 b | 8.79±1.70 bc | 18.53±4.39 a | 0.71±0.18 a |
| LTCF1 | 41.32±11.90 bc | 53.95±12.95 a | 2.15±0.87 c | 2.31±0.73 b | 0.19±0.09 a |
| LTMF1 | 74.45±4.94 a | 15.40±7.05 b | 6.33±2.31 c | 3.73±1.87 ab | 0.03±0.02 a |
| LTBF2 | 82.55±3.49 a | 0.01±0.00 b | 7.52±2.59 bc | 9.10±3.72 ab | 0.80±0.25 a |
| LTCF2 | 85.83±2.94 a | 0.01±0.00 b | 3.88±1.51 c | 10.18±3.54 ab | 0.09±0.03 a |
| LTMF2 | 64.30±6.15 abc | 0.00±0.00 b | 31.54±5.58 a | 3.56±1.22 ab | 0.30±0.14 a |
| LTBF3 | 74.20±8.98 a | 0.01±0.00 b | 6.68±3.06 bc | 17.16±6.01 ab | 1.87±1.14 a |
| LTCF3 | 36.41±3.80 c | 50.00±6.16 a | 6.62±1.60 bc | 5.31±1.37 ab | 1.51±0.80 a |
| LTMF3 | 59.31±6.99 abc | 10.05±5.53 b | 19.27±2.56 ab | 10.33±4.15 ab | 1.01±0.54 a |

a) Mean±SE. Means within the same column followed by different letters are significantly different at *P* ≤ 0.05, as determined by an ANOVA: Tukey test.

**Supplementary table 9** Effect of Host Shift on the top 5 Gut Microbes of the Loofah Population of *B. cucurbitae* at the genus level

| Treatment | *Providencia*(%) | *Morganella*(%) | *Campylobacter*(%) | *Ralstonia*(%) | *Lactococcus*(%) |
| --- | --- | --- | --- | --- | --- |
| LTBF1 | 12.72±6.35 b | 1.07±0.92 a | 0.71±0.48 b | 7.01±5.30 b | 13.86±3.41 a |
| LTCF1 | 7.57±3.10 b | 0.42±0.12 a | 53.93±12.95 a | 8.07±2.14 b | 1.75±0.69 b |
| LTMF1 | 9.35±7.31 b | 18.12±9.51 a | 15.30±7.08 b | 28.09±8.41 a | 2.28±1.33 ab |
| LTBF2 | 10.64±8.12 b | 0.02±0.02 a | 0.01±0.00 b | 0 b | 6.71±4.13 ab |
| LTCF2 | 44.64±33.44 a | 10.81±9.68 a | 0.00±0.00 b | 0 b | 9.19±3.53 ab |
| LTMF2 | 11.47±3.63 b | 11.58±10.18 a | 0.00±0.00 b | 0 b | 1.86±0.95 b |
| LTBF3 | 31.03±25.00 ab | 0.35±0.34 a | 0.01±0.00 b | 0 b | 6.38±3.43 ab |
| LTCF3 | 6.74±3.60 b | 1.31±0.59 a | 50.00±6.16 a | 0 b | 2.77±0.94 ab |
| LTMF3 | 13.70±11.00 ab | 7.28±2.74 a | 9.80±5.63 b | 0 b | 0.62±0.26 b |

a) Mean±SE. Means within the same column followed by different letters are significantly different at *P* ≤ 0.05, as determined by an ANOVA: Tukey test.

**Supplementary table 10** Effect of Host Shift on the top 5 Gut Microbes of the Bitter Gourd Population of *B. cucurbitae* at the phylum level

| Treatment | Proteobacteria(%) | Epsilonbacteraeota(%) | Bacteroidetes(%) | Firmicutes(%) | Actinobacteria(%) |
| --- | --- | --- | --- | --- | --- |
| MTBF1 | 89.57±2.80 a | 0.04±0.01 b | 6.52±1.80 a | 3.68±0.98 a | 0.06±0.02 a |
| MTCF1 | 72.12±11.48 ab | 20.34±11.78 ab | 3.54±1.48 a | 3.82±2.07 a | 0.10±0.01 a |
| MTLF1 | 59.18±10.93 ab | 15.44±9.25 ab | 10.78±4.58 a | 14.27±2.72 a | 0.31±0.12 a |
| MTBF2 | 51.90±9.95 b | 40.69±11.38 a | 1.34±0.53 a | 5.79±2.12 a | 0.14±0.09 a |
| MTCF2 | 65.63±12.85 ab | 0.25±0.23 b | 13.29±4.75 a | 20.49±13.55 a | 0.33±0.16 a |
| MTLF2 | 61.02±2.75 ab | 20.33±5.27 ab | 9.34±3.86 a | 9.07±2.50 a | 0.19±0.08 a |
| MTBF3 | 59.68±2.58 ab | 19.74±3.80 ab | 9.18±2.45 a | 9.62±6.44 a | 1.65±1.17 a |
| MTCF3 | 76.55±4.57 ab | 0.85±0.63 b | 9.05±3.72 a | 12.60±4.63 a | 0.82±0.42 a |
| MTLF3 | 81.56±4.03 ab | 0.09±0.09 b | 12.02±2.46 a | 4.64±3.12 a | 1.35±0.96 a |

a) Mean±SE. Means within the same column followed by different letters are significantly different at *P* ≤ 0.05, as determined by an ANOVA: Tukey test.

**Supplementary table 11** Effect of Host Shift on the top 5 Gut Microbes of the Bitter Gourd Population of *B. cucurbitae* at the genus level

| Treatment | *Providencia*(%) | *Morganella*(%) | *Campylobacter*(%) | *Ralstonia*(%) | *Lactococcus*(%) |
| --- | --- | --- | --- | --- | --- |
| MTBF1 | 10.60±2.39 b | 25.81±16.40 a | 0.02±0.01 b | 19.94±5.65 a | 2.86±0.84 a |
| MTCF1 | 35.31±11.20 ab | 2.71±1.07 a | 19.65±11.86 ab | 6.61±1.53 bc | 1.86±0.72 a |
| MTLF1 | 26.37±15.36 ab | 3.69±2.75 a | 15.43±9.24 ab | 14.11±4.18 ab | 12.16±2.35 a |
| MTBF2 | 24.55±8.15 ab | 19.47±16.61 a | 40.69±11.38 a | 0 c | 4.03±2.06 a |
| MTCF2 | 6.79±2.67 b | 4.99±3.84 a | 0.25±0.23 b | 0 c | 18.60±13.47 a |
| MTLF2 | 24.18±6.47 ab | 9.69±6.89 a | 20.33±5.27 ab | 0 c | 6.65±2.22 a |
| MTBF3 | 31.06±7.74 ab | 14.26±8.94 a | 19.74±3.80 ab | 0 c | 8.84±6.53 a |
| MTCF3 | 35.58±10.92 ab | 13.28±11.15 a | 0.21±0.13 b | 0 c | 8.98±4.60 a |
| MTLF3 | 58.99±7.86 a | 1.51±0.92 a | 0.03±0.02 b | 0 c | 0.30±0.22 a |

a) Mean±SE. Means within the same column followed by different letters are significantly different at *P* ≤ 0.05, as determined by an ANOVA: Tukey test.
