# Supplementary figures and images for: Effect of host shift on the gut microbes of Bactrocera cucurbitae (Coquillett) (Diptera: Tephritidae)
Source: Front Microbiol. 2023 Nov 21;14:1264788. doi: 10.3389/fmicb.2023.1264788 (PMC10703373; doi:10.3389/fmicb.2023.1264788)

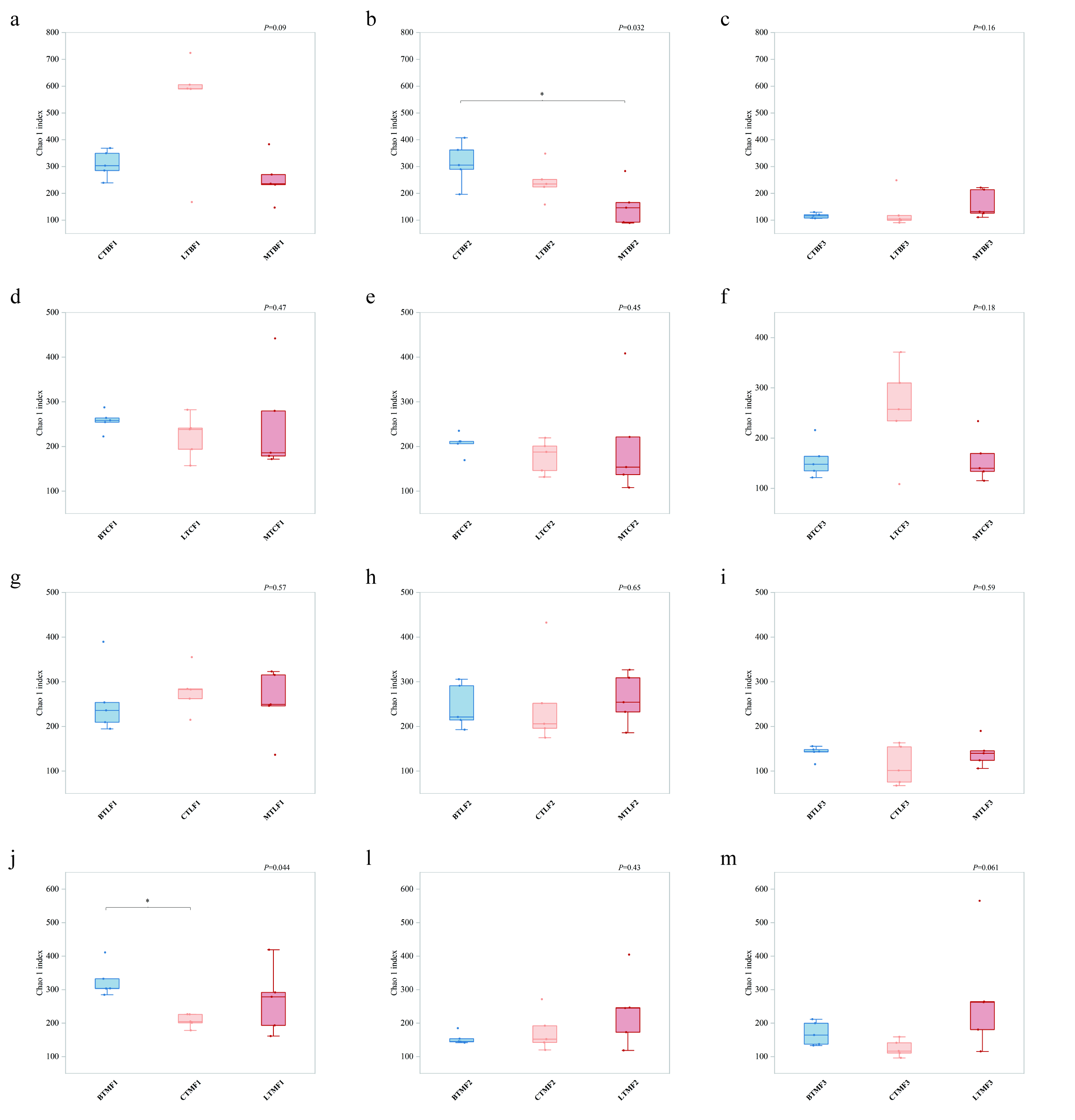

Supplement: Supplementary Figure S1 — Changes in Chao1 index (±SE) after host shift of Bactrocera cucurbitae. [file Image_1.TIF]

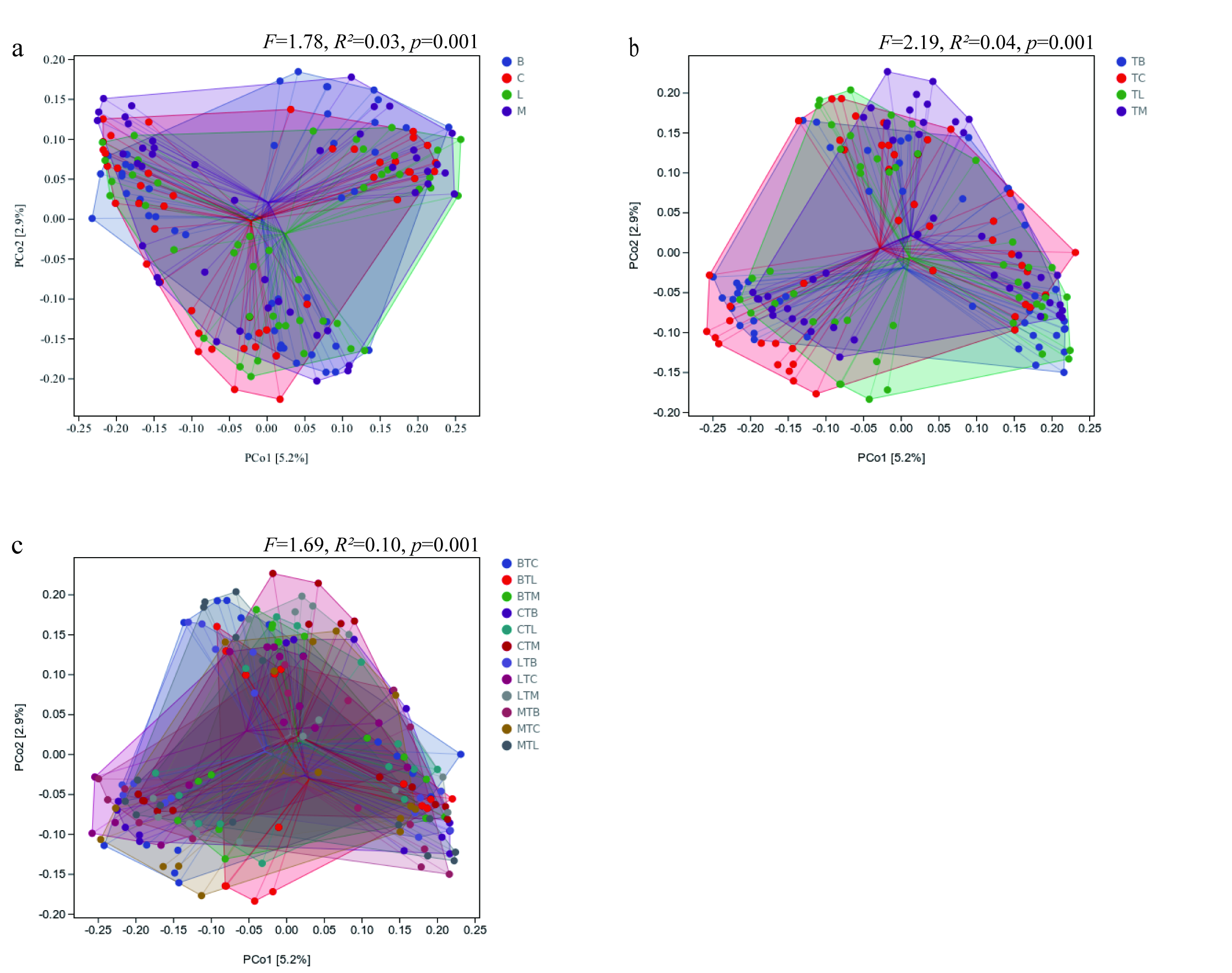

Supplement: Supplementary Figure S2 — Principal coordinate analysis of the microbiota communities of Bactrocera cucurbitae after host shift based on Jaccard distance. [file Image_2.TIF]

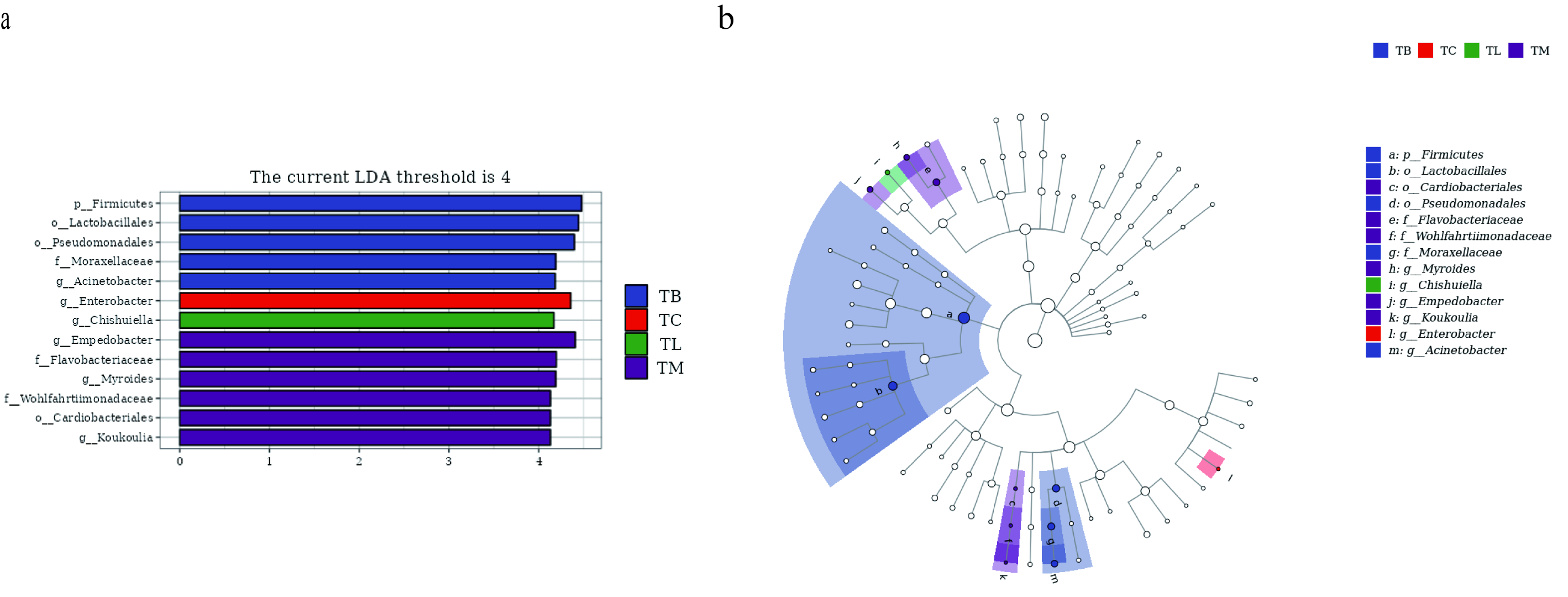

Supplement: Supplementary Figure S3 — Bacterial taxa with LDA scores of host shift of the Bactrocera cucurbitae, from the phylum to genus levels, with an LDA score >2. [file Image_3.TIF]

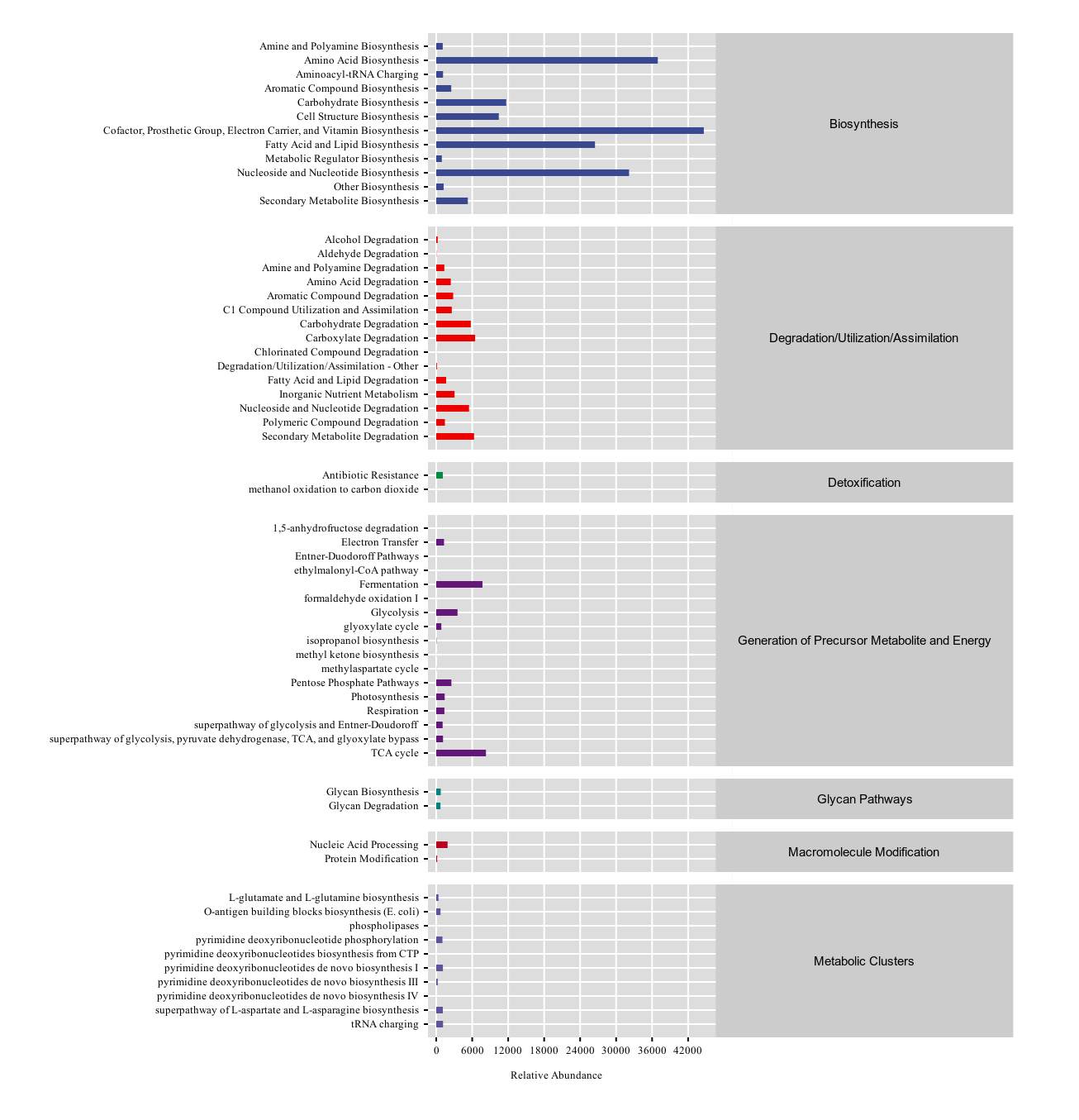

Supplement: Supplementary Figure S4 — Comparison of predicted GO functions of the gut bacteria of Bactrocera cucurbitae after host shift. [file Image_4.PNG]

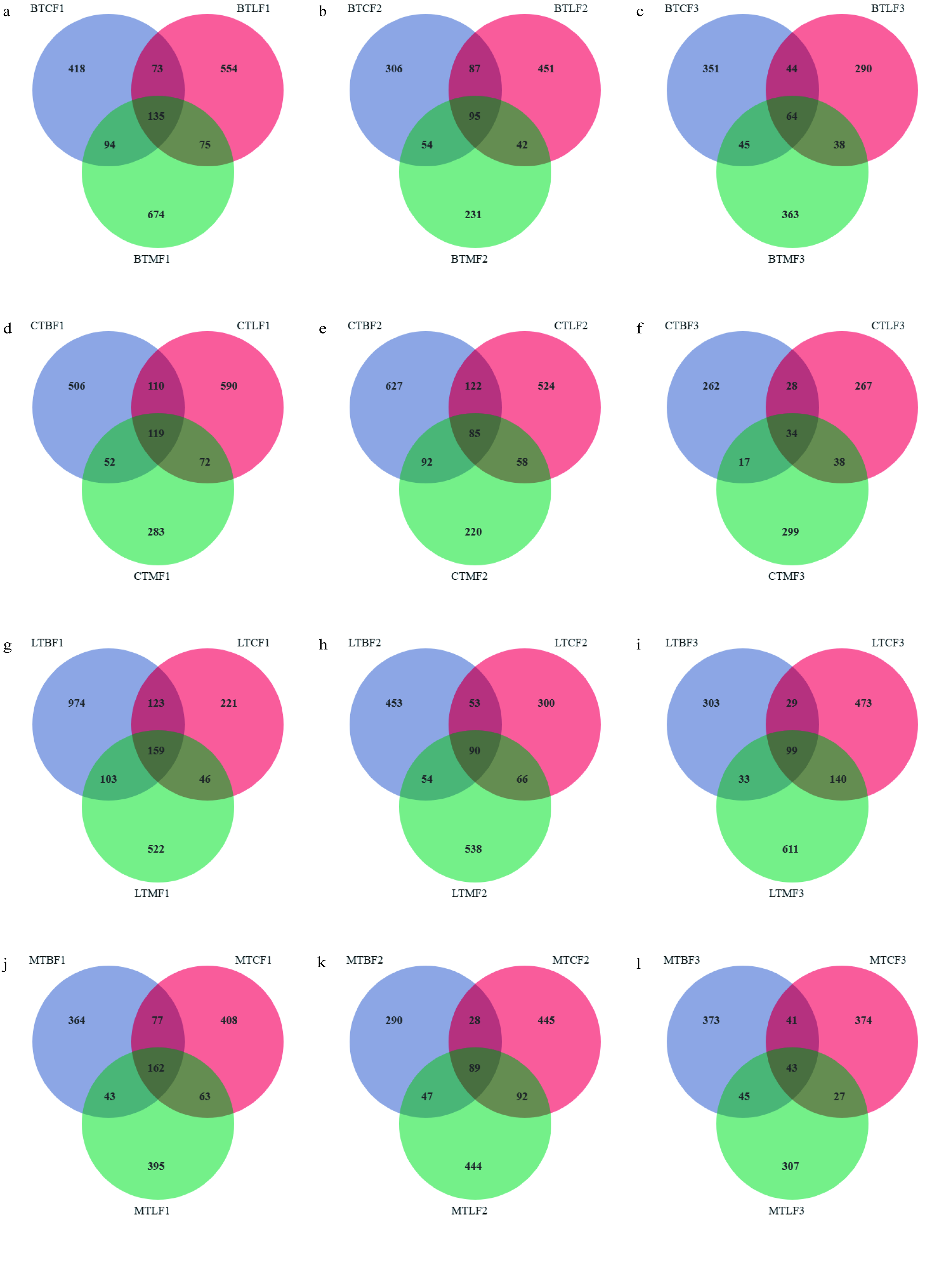

Supplement: Supplementary Figure S5 — Venn diagram of microbiota community OTUs of Bactrocera cucurbitae after host shift. [file Image_5.TIF]

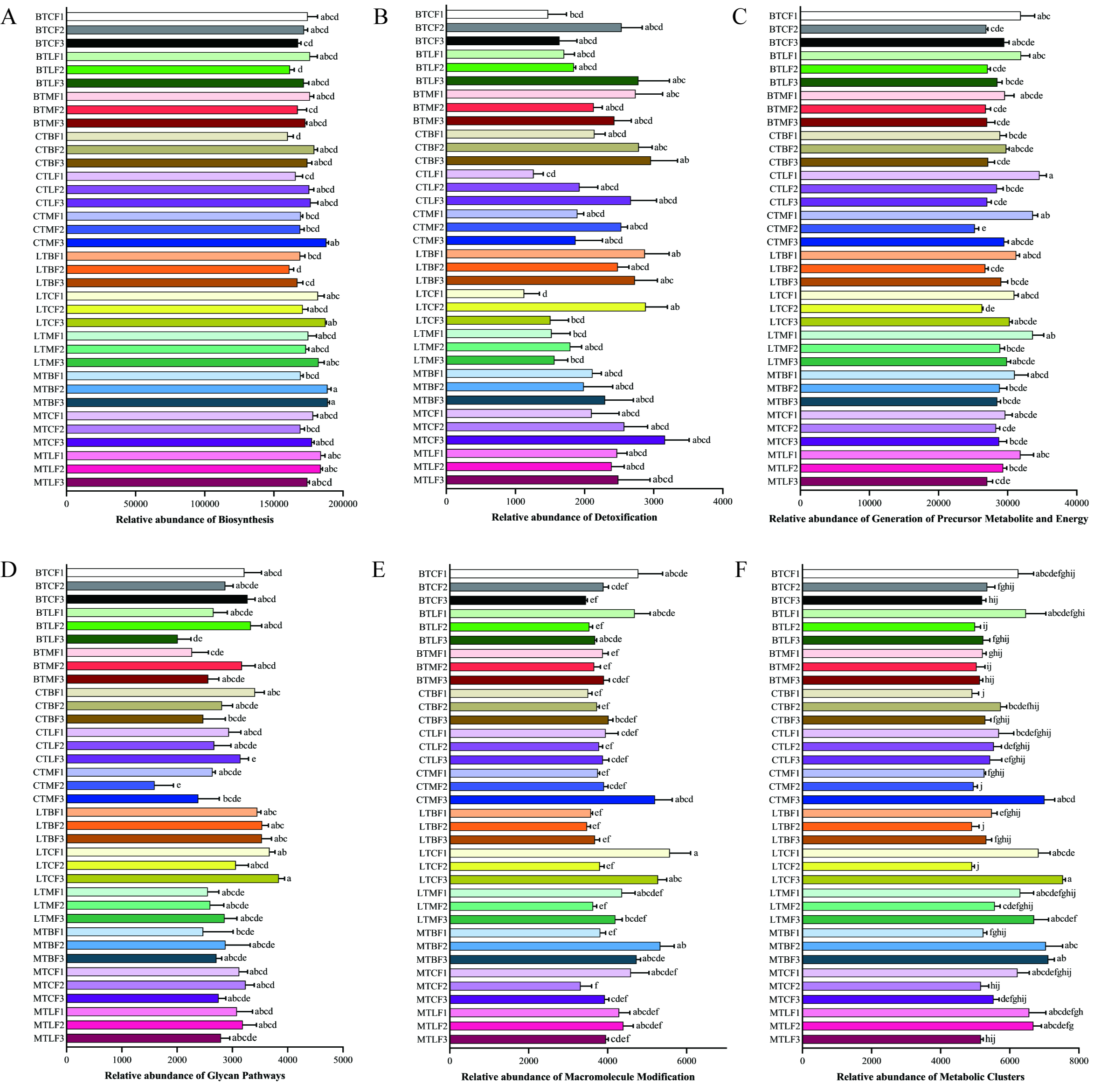

Supplement: Supplementary Figure S6 — Comparison of predicted GO functions of the gut bacteria of Bactrocera cucurbitae after host shift. [file Image_6.TIF]
